# The Draft Genome and Transcriptome of the Atlantic Horseshoe Crab,* Limulus polyphemus*

**DOI:** 10.1155/2017/7636513

**Published:** 2017-02-06

**Authors:** Stephen D. Simpson, Jordan S. Ramsdell, Winsor H. Watson III, Christopher C. Chabot

**Affiliations:** ^1^Hubbard Center for Genome Studies, University of New Hampshire, Durham, NH 03824, USA; ^2^Department of Biological Sciences, University of New Hampshire, Durham, NH 03824, USA; ^3^Department of Biological Sciences, MSC#64, Plymouth State University, Plymouth, NH 03264, USA

## Abstract

The horseshoe crab,* Limulus polyphemus*, exhibits robust circadian and circatidal rhythms, but little is known about the molecular mechanisms underlying those rhythms. In this study, horseshoe crabs were collected during the day and night as well as high and low tides, and their muscle and central nervous system tissues were processed for genome and transcriptome sequencing, respectively. The genome assembly resulted in 7.4 × 10^5^ contigs with N50 of 4,736, while the transcriptome assembly resulted in 9.3 × 10^4^ contigs and N50 of 3,497. Analysis of functional completeness by the identification of putative universal orthologs suggests that the transcriptome has three times more total expected orthologs than the genome. Interestingly, RNA-Seq analysis indicated no statistically significant changes in expression level for any circadian core or accessory gene, but there was significant cycling of several noncircadian transcripts. Overall, these assemblies provide a resource to investigate the* Limulus* clock systems and provide a large dataset for further exploration into the taxonomy and biology of the Atlantic horseshoe crab.

## 1. Introduction

The Atlantic horseshoe crab,* Limulus polyphemus*, is an important species for a variety of reasons. First, it is considered a keystone species within many estuaries and bays along the Atlantic coast of the United States. Moreover, in these habitats their foraging behavior releases trapped nutrients into their local environment [1, 2]. Second, their eggs are a source of food for endemic and endangered long distance avian migrants. Third, they are used as bait for the eel and conch fisheries [3]. Fourth, their blood is the source of the most sensitive assay for gram-negative bacteria contamination of medical products [4–6]. Finally,* Limulus* have long been used as model systems in neurobiological research. For example, lateral inhibition, one of the underlying principles of visual physiology, was first demonstrated in* Limulus* by Haldan Keffer Hartline, and as a result he won the Nobel Prize in Physiology or Medicine in 1967 [7]. More recently, this species has been used as a model for the investigation biological rhythms, for instance, circadian rhythms of visual sensitivity [8–10].

Circadian rhythms are produced by a combination of endogenous clocks and cues from the environment [11, 12]. The identity of several core circadian genes that are responsible for producing these rhythms has been revealed in a number of invertebrates and vertebrates [13] and includes* period*,* clock*,* cryptochrome*,* cycle*,* timeless,* and other accessory genes. Although these genes are labeled as circadian, due to their critical function within the circadian clock mechanism, they may also play a role in other types of biological rhythms including those that regulate seasonal activity [14]. It has also been proposed, but not demonstrated, that they might be involved in shorter (~12.5 hr) circatidal rhythms [9, 15, 16]. One of the overall goals of this study was to test this hypothesis in horseshoe crabs, which express both a circadian rhythm of lateral eye sensitivity [17] and a circatidal rhythm of locomotion [10, 18].

In this study we developed draft genomic and transcriptomic assemblies for* Limulus polyphemus* and then compared the genes expressed during high and low tides and during the day versus the night. Particular attention was paid to putative core and accessory circadian genes. We identified these and then compared their expression, using RPKM values, across the different conditions (tides and light : dark (L : D)). Because no clear differences in the expression of putative circadian genes were apparent, we further examined some of the transcripts that did exhibit significant day/night or high/low tide differences as a first step towards the identification of potential proteins involved in the temporal control of the behavior and physiology in this species.

## 2. Methods

### 2.1. Animals and Environmental Conditions

For genomic sequencing, an individual horseshoe crab was wild-caught from Great Bay Estuary in Durham, NH (43°05′30′′N and 70°51′55′′W). Leg skeletal muscle tissue was removed and placed in liquid nitrogen for immediate DNA extraction (described in the following).

For transcriptome sequencing, four animals were captured from Great Bay Estuary in Durham, NH, and sacrificed at four different times: day high tide (DHT, 0800), night high tide (NHT, 2030), evening low tide (ELT 1800), and during the day at low tide (DLT, 1530). DLT was collected while still being active (during high tide), placed into a natural water flow-through tank located next to the bay with open exposure, and sacrificed, while being inactive (buried), at the time of low tide (1530). DHT and NHT were used to compare the expression of genes during the day versus the night, and DHT and DLT were used to compare expression during high and low tides. Tissues from ELT were sequenced and used to increase the overall depth of the combined transcriptome dataset. Animals were dissected and their entire central nervous system tissue (protocerebrum, subesophageal ganglia, ventral nerve cord, and ganglia) was snap frozen on dry ice.

### 2.2. DNA Extraction

300 mg of frozen muscle tissue was pulverized using a sterile, autoclaved mortar and pestle. 19 mL of Qiagen G2 lysis buffer (Qiagen #1014636) spiked with 38 *μ*L of Qiagen RNase A stock solution (100 *μ*g/*μ*L, Qiagen #19101) was combined with the ground tissue in a sterile 50 mL conical tube. DNA was extracted per the Qiagen Genomic-Tip 500/G protocol (Qiagen genomic DNA Handbook, 08/2001). Extracted gDNA was stored at −80°C until library preparation.

### 2.3. RNA Extraction for Transcriptome Sequencing

Frozen whole CNS tissue was shipped on dry ice to University of Vermont Cancer Center DNA Analysis Facility (Burlington, VT) for extraction. Tissue was stored at −80°C until RNA extraction. Frozen tissue was homogenized in 1 mL of Ambion® TRIzol® Reagent (LifeTech, #15596-026) using a sterile, autoclaved mortar, and pestle. RNA was extracted per the Ambion® TRIzol® protocol (LifeTech, MAN0001271) and purified using Qiagen RNeasy Mini Kit (Qiagen #74104) per manufacturers protocol (Qiagen RNeasy Mini Handbook, 06/2012).

### 2.4. Genome Illumina Paired-End Library Construction and Sequencing

Extracted gDNA was sent to Vanderbilt Technologies and Advanced Genomics Facility, Nashville, TN. The library was prepared using TruSeq DNA Sample Preparation Guide v2, catalog #FC-930-1021 (Illumina, San Diego, CA, USA). One g of gDNA was sheared using a Covaris S2. Sheared ends were then repaired and adenylated and the products were ligated with Illumina adaptors. Ligated fragments were then size selected using Pippen Prep (Sage Science, Beverly, MA, USA) and cleaned using Zymo Clean and Concentrator Kit (Zymo Research, Irvine, CA, USA). KAPA Hot Start (KAPA Biosystems, Wilmington, MA, USA) was then used the amplify libraries over a total of 14 PCR cycles. Clustering was performed using a cBot (Illumina) and paired-end sequencing was performed on a HiSeq 2000 (Illumina) over two lanes.

### 2.5. Transcriptome Illumina Paired-End Library Construction and Sequencing

Extracted, purified RNA was quantified and analyzed using a Qubit 2.0 (Life Technology, Carlsbad CA) and an Agilent Bioanalyzer 2100 (Agilent Technology, Santa Clara, CA). RNA libraries were prepared using Illumina TruSeq RNA Sample Prep LT version 2 (RS-122-2001/2002). 1 *μ*g of total RNA was PolyA enriched using AMPure XP Magnetic Beads (Beckman Coulter #A63880). Complementary DNA libraries were created from the enriched RNA using Invitrogen Superscript II® per the manufacturers protocol. cDNA was fragmented, end repaired, and adenylated followed by a ligation of Illumina adaptor indices. Illumina Reagents (Part #15012995) were used for PCR amplification. TruSeq libraries were quantified using Nanodrop, Qubit, and qPCR (KAPA Biosciences kit #4824). Fragment size was determined using Agilent Bioanalyzer 2100 and clustering was performed on the Illumina cBOT. Sequencing was carried out at 12 pM on an Illumina HiSeq1000/1500.

### 2.6. Genome De Novo Assembly

Illumina CASAVA pipeline was used to demultiplex and filter* Limulus* genomic reads (reads with *Q* < 10 were removed). Reads were assembled in CLC Genomics Workbench (v 5.1.2.) using CLC Bio's Proprietary CLC Assembly Cell 4.0 (CLC4) set to default parameters at the Hubbard Center for Genome Studies at the University of New Hampshire (Durham, NH).

### 2.7. Transcriptome De Novo Assembly

Four unique conditions (DHT, NHT, ELT, and DLT) were assembled separately in CLC Genomics Workbench (v 5.1.2.) using CLC Bio's Proprietary CLC Assembly Cell 4.0 (CLC4) set to default parameters. Additionally, all four conditions were combined and again assembled using the same algorithm and parameters for use as a reference library.

### 2.8. Benchmarking Universal Single-Copy Orthologs (BUSCO) Analysis

Genome and transcriptome completeness's were assessed using BUSCO v1.1 using the eukaryotic linage for both and default parameters for the genome and transcriptome analyses, respectively.

### 2.9. mtDNA Analysis

**Figure 1:**
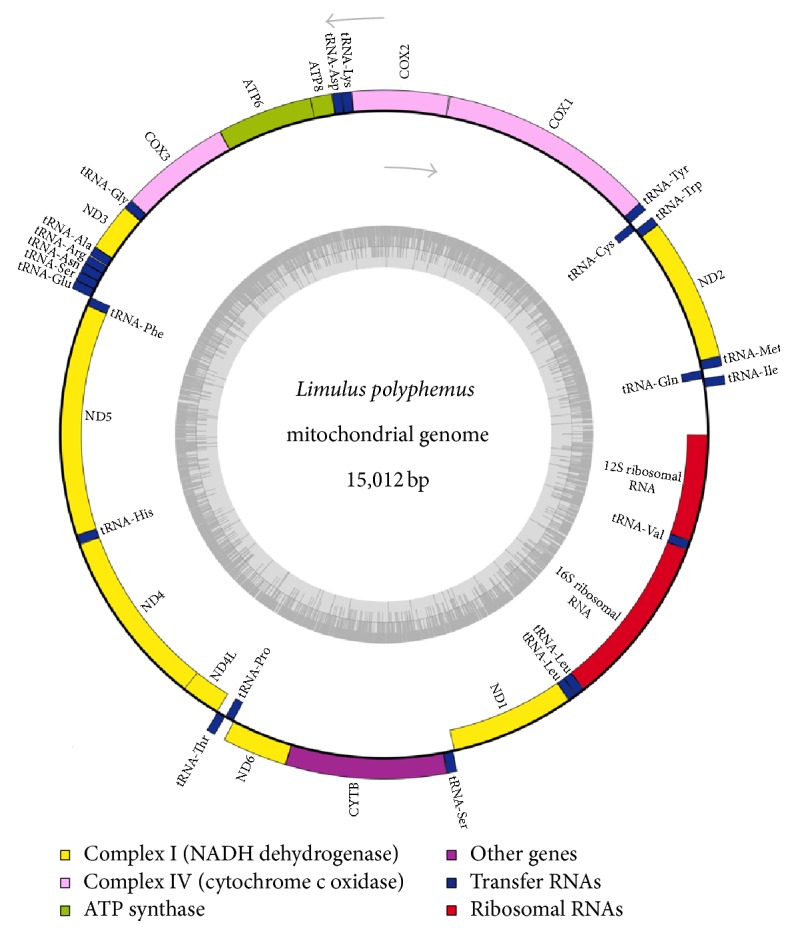
Gene map of the* Limulus polyphemus* mitochondrial genome. Arrows indicate strand direction with the inner circle representing genes on the light strand while the outer circle represents genes on the heavy strand. ND1–6 represents nicotinamide adenine dinucleotide dehydrogenase (NADH) subunit variants. tRNA (“amino acid”) represents transfer RNA variants. COX1–3 represents the cytochrome c oxidase subunits. CYTB represents cytochrome b oxidase. ATP6 and ATP8 represent adenosine triphosphate variants 6 and 8. Rrn12 and rrn16 represent ribosomal subunits 12 and 16. Innermost dark grey circle represents distribution of GC content.

The previously published* Limulus* mitochondrial genome (NC_003057.1) was blasted against the genomic assembly and used to identify* Limulus* genomic contig 669. Contig 669 was then analyzed for coding regions and fully annotated using NC_003057.1 as a reference and visualized (Figure 1) using Organellar Genome DRAW [19].* Limulus* mtDNA was then blasted against the* Limulus* genome assembly to look for nuclear mitochondrial (NUMT) sequences. To validate potential NUMT sequences, genomic contigs that contained homologous regions of mtDNA were extracted and compared for similarity.

### 2.10. Transcriptome Analysis

Individual read sets from the four samples were mapped to the overall transcriptome assembly, with each contig given a unique identifying number. Reads per kilobase per million mapped reads (RPKMs) were used to determine relative fold change between day/night and high/low tides. Blast2GO [20] was performed on each of the four RNA-Seq mappings to look for expression variation in molecular groupings. Transcripts between conditions were normalized by identifying the condition with the highest number of sequences across all defined gene ontologies (GO) and dividing that condition's GO sequence numbers by the corresponding GOs of another condition marked for comparison. The average difference between GOs of the two individuals (to be compared) was then used as a normalization value for the actual sequence number of the individual with less sequences per GO.

### 2.11. Accession Numbers

*Limulus* genome and transcriptome sequence read data are located in the NCBI Sequence Read Archive under accession numbers [SRR4181534] and [SSR421559-SSR4215562], respectively. The* Limulus* mtDNA genome sequence is located on NCBI under the accession number JX983598.

## 3. Results

### 3.1. *Limulus* Genome Sequencing and Assembly

Genomic sequences yielded a total of 620,743,244 one hundred base pair (paired-end) reads (Table 1). The* Limulus* genome size is estimated to be 2.74 Gb based on biochemical analysis [21], and, based on that estimation, these sequences should result in an approximate 23x coverage of the genome (Table 1). De novo assembly of the genome resulted in N50 of 4.7 kb with the largest contig being 68.9 kb. The total length of all contigs ≥ 500 bp was 1.4 Gb. To evaluate the completeness of the genome assembly with respect to protein coding genes, BUSCO v1.1 genome assessment was used to look for evidence of 429 putative universal orthologs and yielded the following results: complete genes found (C), 12.9%; duplicated genes found (D), 1.6%; fragmented genes found (F), 10.7%; genes missing (M), 74.8%; number of genes used (N), 429. Overall only 25.2% of the genes that were looked for were found within the genome [22].

### 3.2. *Limulus* mtDNA

The overall length of the* Limulus* mitochondrial genome is 15,012 bp, which is longer than the other previously published* Limulus* mitochondrial genome at 14,985 bp [23]. The majority of extra mtDNA appears to be in the noncoding origin of the mtDNA genome. However, there are a number of polymorphisms, insertions, and deletions between the mitochondrial genome sequences, many of which occur in the coding regions (Table 2).

### 3.3. *Limulus* NUMT Sequences

**Figure 2:**
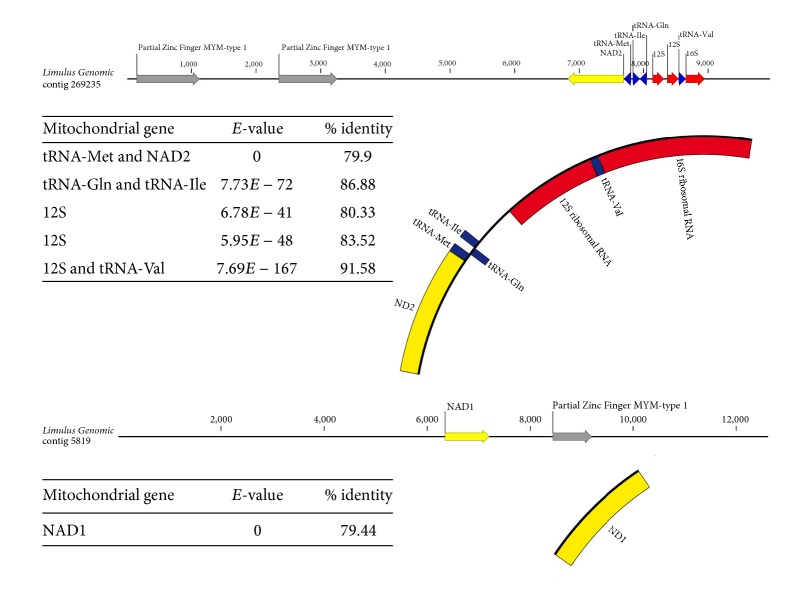
Diagram of two nuclear mitochondrial (NUMT) sequences identified in the* Limulus* genome.* Limulus* genomic contigs 269235 and 5819 show the length of each contig, the location of the NUMT sequences (yellow = NAD variants, blue = tRNAs, and red = rRNA), and relative location of* Limulus* genomic sequences (grey = Zinc Finger MYM-type 1). The mitochondrial gene names are given along with the *e*-values and percent identities of the mitochondrial sequences to the NUMT sequences.

Two NUMT sequences were identified as genomic contigs 269235 and 5819, which were 9,955 bp and 12,620 bp in length, respectively. These two nuclear contigs each had significant stretches of sequence that were clearly homologous to the extant mitochondrial genome. Specifically, nuclear contig 269235 contained a contiguous region of 2,008 bp that includes sequences homologous to the mitochondrial genes for* nad2*,* methionyl-tRNA*,* lysidine synthase*,* glutamate-tRNA ligase*,* valine-tRNA ligase*, and* 12s ribosomal RNA* and contig 5819 includes 1,065 bp homologous to* nad1*. To confirm that the NUMT sequences were not simply errors in assembly, we compared the NUMTs and extant mitochondrial sequences. Neither of the NUMT sequences in genomic contigs 269235 or 5819 should have high levels of sequence identity expected if they were assembled from extant mitochondrial genome reads. Specifically NUMT sequences in genomic contig 269235 had an 84.4% average percent identity to mtDNA sequences and those in genomic contig 5819 had a 79.4% identity to the homologous mtDNA sequences (Figure 2).

### 3.4. *Limulus* Transcriptome Sequencing and Assembly

**Figure 3:**
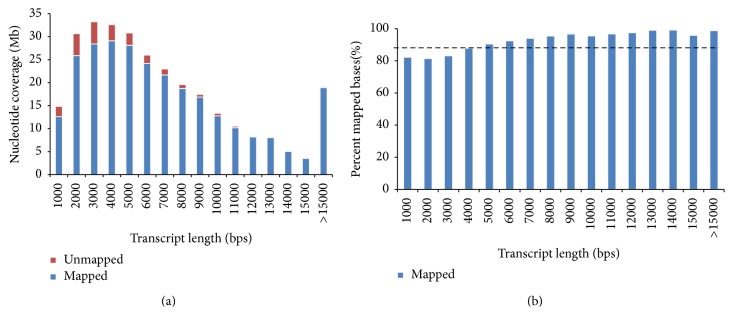
Transcriptome assembly mapped to the genome assembly. (a) Nucleotide coverage: *y*-axis: total number of nucleotides that could be mapped for each transcript length bin (1000 nucleotides). (b) Percent mapped bases: *y*-axis: total percentage of mapped bases in each bin. Black dashed line: the percentage of nucleotides in the transcriptome that could be mapped back to the genome.

The combined dataset had a total of 484,378,406 reads at a size of 100 bp and was de novo assembled in CLC to generate a reference assembly for analysis. The resulting transcriptomic assembly contained 92,348 contigs with a maximum contig size of 23.85 kb. Transcripts mapped back to the genome with 88.3% of the total transcripts, equating to 109.76 Mb, suggesting that the transcriptome assembly represents most of the exons present in our genome assembly (Figure 3). The completeness of the transcriptome was evaluated using BUSCO v1.1 transcriptome assessment to look for evidence of 429 putative universal orthologs, which yielded the following results: complete genes found (C), 56.6%; duplicated genes found (D), 10.7%; fragmented genes found (F), 13.3%; genes missing (M), 19.3%. Overall the transcriptome was found to have 80.7% of the total number of genes looked for in the BUSCO analysis, more than three times that of the genome [22]. Sequencing and assembly statistics obtained for the genomic and transcriptomic libraries are summarized in Table 1.

### 3.5. Effects of Photoperiod and Tidal Phase on* Limulus* Transcript Expression

**Figure 4:**
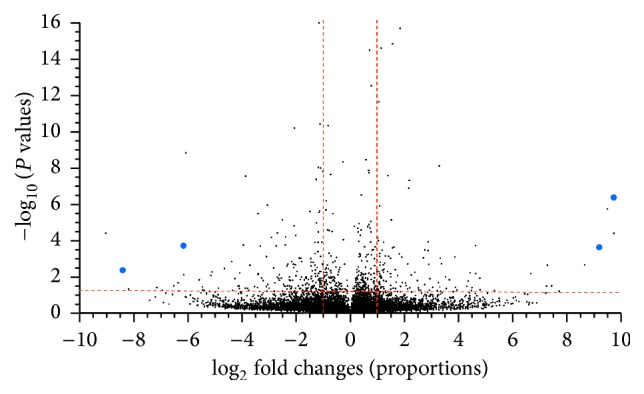
Volcano plot of day versus night showing the distribution of transcripts with respect to fold change (*x*-axis) and statistical significance (*y*-axis). Points above the horizontal red line indicate transcripts whose abundance is statistically significant (*P* value ≤ 0.05). Points to the left of −1 log_2_ fold change and the right of +1 log_2_ fold change indicate transcripts with at least a doubling in abundance relative to day and night. Large blue dots indicate top four contigs chosen for analysis in Table 3.

**Figure 5:**
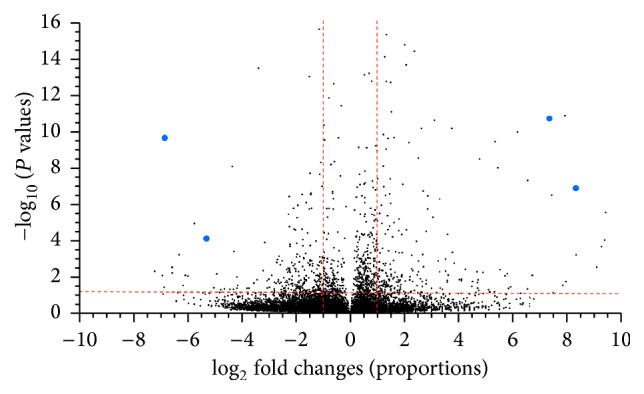
Volcano plot of high versus low tide showing the distribution of transcripts with respect to fold change and statistical significance.

**Figure 6:**
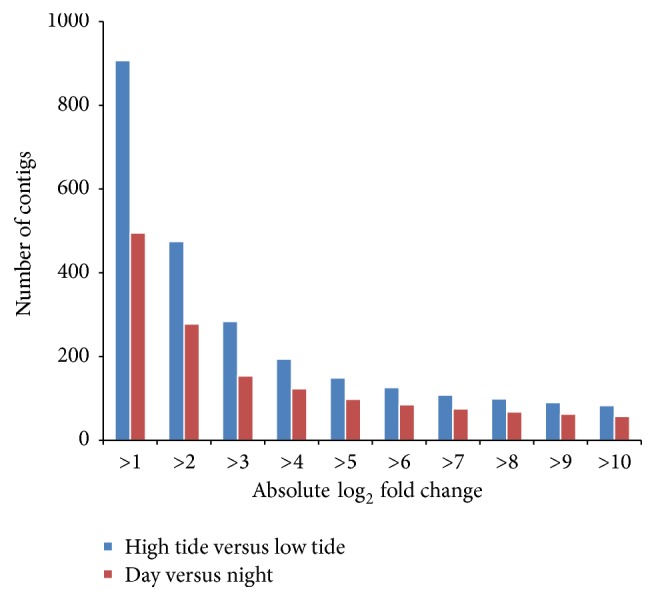
Number of statistically significant (*P* ≤ 0.05) transcripts (contigs) in both the day/night (red bars) and high tide/low tide (blue bars) volcano plots grouped by Log_2_ fold change. Results indicate a greater presence of statistically significant transcripts for tidal phase relative to photoperiod.

The primary purpose of the RNA-Seq data was to generate a central nervous system transcriptome, while a secondary purpose was to generate a set of pilot data for potential differences with regard to photoperiod and tidal phase. Of the four conditions used for transcriptomic analysis (DHT, NHT, ELT, and DLT), three were used for the following comparisons: DHT/NHT and NHT/DLT. Total read counts for each condition were 1.5 × 10^8^ for DHT, 1.3 × 10^8^ for NHT, 5.8 × 10^7^ for ELT, and 1.4 × 10^8^ for DLT. To compare gene expression across the different treatments we mapped the reads from each dataset to the combined reference assembly using CLC Genomics Workbench's (v5.1.2) RNA-Seq analysis toolkit. Volcano plot analysis (CLC v5.1.2) was used to analyze variation between night and day samples as well as high and low tide samples (Figures 4 and 5). As expected, the majority of sequences for both conditions fell below the thresholds of statistical significance (below horizontal red line) and a minimum fold change of 2 (in between vertical red lines). However, when the number of contigs that varied significantly was compared between the two treatments (Figure 6), there was a larger number of contigs with a minimum fold change of ≥2 that varied significantly in the high/low tide comparison than in the day/night comparison.

### 3.6. Expression of Gene Types with respect to Photoperiod and Tidal Phase

The majority of transcripts that were different in the photoperiod and tidal phase comparisons were novel [24]. Therefore, only the top four identifiable genes with the lowest *P* values and highest fold changes are shown for both comparisons in Table 3. Of the genes that could be identified when BLASTed against NCBI, twice as many were affected by tidal versus photoperiodic conditions (*e*-values <1.0*E* − 4; Table 3). Additionally, genes with *e*-values <1.0*E* − 22 were primarily developmental and regulatory in nature (Table 3).

**Figure 7:**
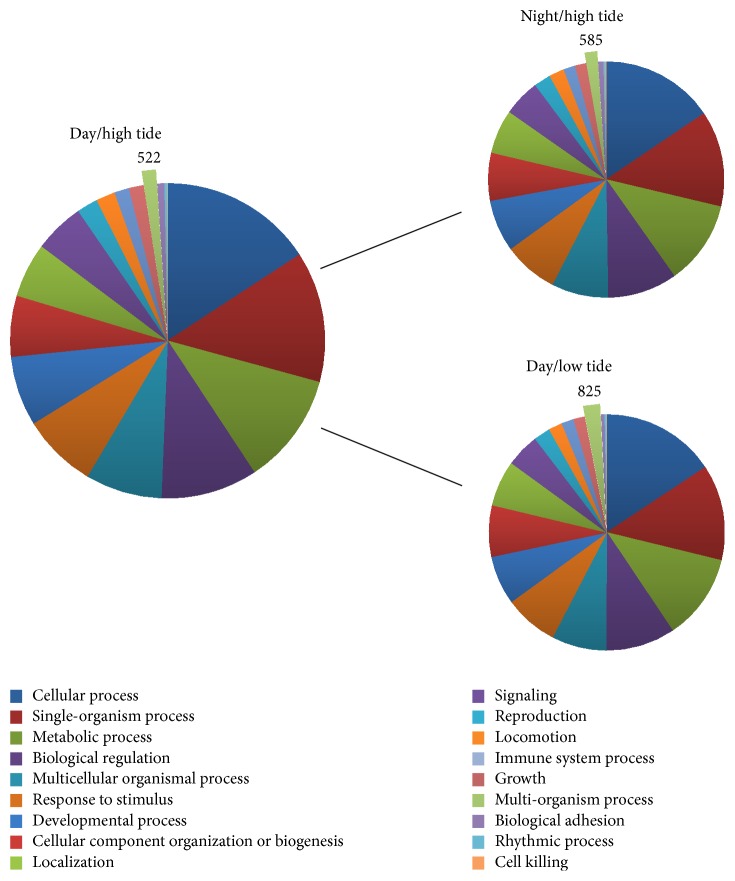
Biological process BLAST2GO values for RNA-Seq conditions: day/high tide and night and low tide. Data labels are shown in conjunction with their respective slice of the pie graph. Enlarged slices represent GO values with a percent change greater than 40%.

**Figure 8:**
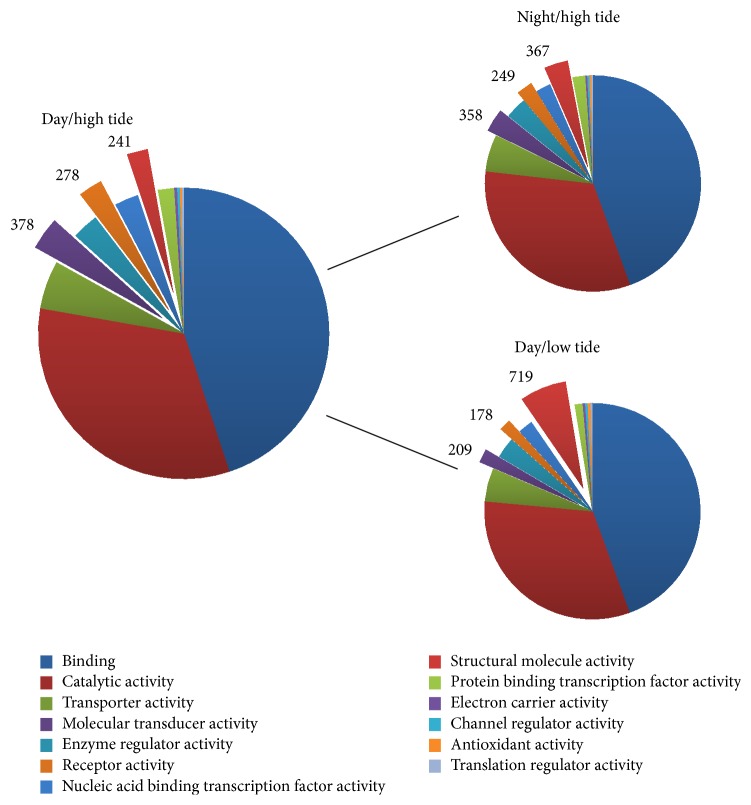
Molecular function BLAST2GO values for RNA-Seq conditions: day/high tide and night and low tide.

**Figure 9:**
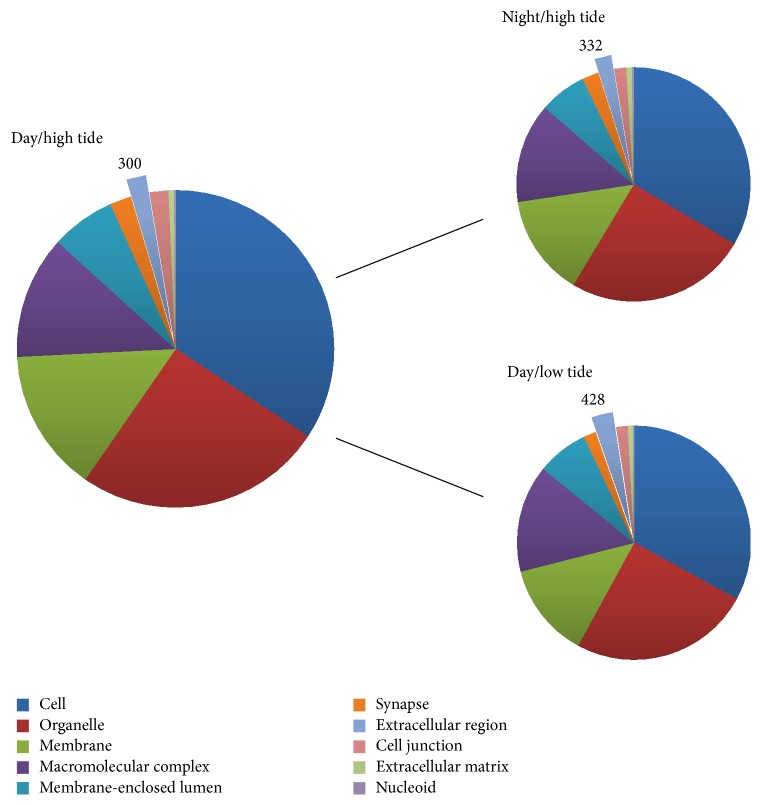
Cellular component BLAST2GO values for RNA-Seq conditions: day/high tide and night and low tide.

All transcript contigs from photoperiodic and tidal conditions were also annotated using the BLAST2GO pipeline, which uses gene ontology (GO) to define genes based on common functions and relationships. The GO terms “multiorganism process” (Figure 7), “molecular transducer activity,” “receptor activity,” “structural molecule activity” (Figure 8), and “translation regulator activity” (Figure 9) all varied greater than 40% when looking at the comparison between high and low tides. Only 1 gene ontology term, “structural molecule activity,” appeared to change more than 40% when looking at night versus day (Figure 8).

### 3.7. Targeted Analysis of Candidates Genes Involved in Circadian Pathways in Drosophila

Candidate genes homologous to* Drosophila* were selected as they are considered the core circadian genes from the model insect organism,* Drosophila melanogaster*.* Cycle *1 (KX014724) RPKM values had the largest absolute fold change with respect to tidal phase with a value of 4.3-fold (Table 4). Interestingly,* cycle 1* was also among the genes with the least amount of variance when looking at differences in photoperiod (1.1-fold).* Cycle 2* (KX014725), however, has approximately the same amount of change when looking at photoperiod or tidal phase (3.1-fold and 3.3-fold, respectively).* Cryptochrome 1*,* cryptochrome *2 (KX014723),* timeless* (KX014719),* clock* (KX014718), and* period* variants* A* (KX014720),* B* (KX014721), and* C* (KX014722) all had absolute fold change values <2.0 for both conditions of photoperiod and tidal phase.

Relative RPKM values were also calculated for a number of circadian accessory genes [25–28] and potential control genes [29–32], as shown in Table 4. The accessory genes* timeout*,* double-time*,* vrille* (KX014726), and* casein kinase*'s* Iα* (KX014742),* IIα* (KX014727),* IIβ* (KX014741), and* Iε* (KX014743) show an absolute fold change of ≤1.1-fold in all but two cases:* vrille*, with respect to photoperiod (1.6-fold), and* timeout,* with respect to tidal phase (1.9-fold). Additionally, 12 potential control genes were investigated:* C-reactive protein* (CRP),* tubulin* (TUB),* ubiquitin* (UBQ),* succinate dehydrogenase complex*,* subunit A* (SDHA),* synaptotagmin* (SYN), and seven variations of* actin* (ACT). All of the investigated control genes with the exception of the* actin* variants had an absolute fold change ≤1.2-fold for either condition. Notably, six of seven* actin* variations, a gene traditionally used for normalization in QPCR analysis, exhibit an absolute fold change ≥1.3-fold over tidal conditions and four of seven display a variation ≥1.1-fold over photoperiodic conditions. The neuropeptide, pigment-dispersing hormone (PDH), was also investigated as it has been described as a potential downstream regulator of circadian rhythms in arthropods [33, 34]. A PDH receptor-like homolog was identified in the* Limulus* transcriptome dataset and found to have an absolute fold change of 3.0-fold for conditions of tidal phase, whereas the absolute fold change for conditions of photoperiod was only 1.5-fold (Table 4).

## 4. Discussion

Based on the fossil record, the Atlantic horseshoe crab,* Limulus polyphemus*, is one of the most ancient living organisms [35] and therefore the genome and transcriptome presented here will likely both provide clues to evolutionary divergence and lead to the identification of novel genes [36]. The draft sequences for the* Limulus* genome, transcriptome, and the addition of a second mitochondrial sequence will also provide a dataset for mining biomarkers and other molecular tools with which diversity and population studies can be conducted [37]. Thus, these data may also help to provide genetic markers that can be used to identify local subpopulations and lead to improved management of this important species. Finally, the subphylum Chelicerata is underrepresented when it comes to annotated genomes. Currently there is only one other representative from the Chelicerata subphylum which has had its genome sequenced (*Ixodes scapularis* [38]) and so adding a draft genome and transcriptome at 23x coverage and 38.5 Gb, respectively, will greatly increase the genetic reference for all Chelicerata.

*Limulus* has at least two distinct endogenous clocks [8, 9]. There is a circadian clock, which controls day/night sensitivity to light [39] and a clock system that controls circatidal rhythms of locomotor activity [10]. While several core and accessory genes are known to be part of the molecular structure of the circadian clock in model organisms such as* Drosophila* [26–28], the molecular workings of the clock controlling circatidal rhythms are completely unknown [40, 41]. While investigations into the molecular mechanics of this clock in the intertidal mangrove cricket,* Apteronemobius asahinai*, have shown that the circadian genes* period* and* clock* are likely not involved in circatidal rhythms [42, 43], our working hypothesis is that circatidal rhythms evolved from existing circadian mechanisms. Indeed, the findings that circatidal rhythms appear to be controlled by two clocks (“circalunidian clocks” [41, 44]), each of which has an endogenous period (~24.8 h) very close to circadian clocks (~24 h), support this idea. Homologs for all of the core circadian genes have been identified within* Limulus* (Table 4 [45]), two of which (*cycle* and* timeless* variants) seem to vary by time of day and tide (Table 4), supporting the idea that some of core circadian genes are somehow involved in modulating the circadian rhythm as well as the circatidal rhythm.

Overall however, RNA-Seq analysis of the majority of core circadian transcript expression across conditions of photoperiod and tidal phase indicated only slight variation in expression (<2-fold for nearly all) for those conditions. Additionally, QPCR data of the core circadian mRNA within the protocerebrum has shown no statistically significant variation of expression for* clock*,* cycle 1*, or* timeless* (Simpson, unpublished results). When combined, these data suggest a relatively constitutive expression profile when looking at daily and tidal differences of most core circadian clock gene transcripts within the central nervous system as a whole. However, both variants of the core clock gene* cycle* seem to vary moderately (>3-fold) with respect to tidal phase with only one variant (*cycle 2*) varying moderately (>3-fold) with respect to photoperiod. Additionally, investigations into downstream circadian regulators like the homolog of the neuropeptide PDH identified a transcript that is closely related to a PDH receptor but shares a higher percent identity with the vertebrate receptor for corticotropin-releasing hormone (CRH). Changes in RPKM values for the transcript exhibited a much greater change (3-fold) from conditions of tidal phase rather than from conditions of photoperiod (Table 4). Further BLAST investigations of PDH, CRH, and precursors involved with their synthesis yielded no reads [45]. With the majority of investigated circadian transcript expression exhibiting strong tendencies towards tidal phase rather than photoperiod, these findings may provide evidence of genes involved with the* Limulus* circatidal timing system.

When the top 20 most significant transcript contigs were pulled out of the experimental groupings (high tide versus low tide and night versus day) and blasted against the NCBI database, only 20% came back with an identified hit (Table 3). Of the genes that were expressed differentially during conditions of photoperiod, only two had *e*-values < 1.0*E* − 3. The exact function of these genes is not known; however, “DMX-like protein 2” seems to be involved in synaptic processes and “protein trapped in endotherm 1” may play a critical role in germ cell migration. The genes that exhibited the largest changes over conditions of tidal phase all had *e*-values < 1.0*E* − 31. “Thyroid peroxidase-like” and “3 beta-hydroxysteroid dehydrogenase” seem to be involved in hormone production; “ALX homeobox protein 1” is involved with neural development in mice and “ovostatin-like” is similar to a proteinase inhibitor found in chicken eggs (Table 3). The lack of definitive gene identification and the small percentage of transcripts that returned with BLAST evidence of homology suggests that the majority of transcripts found to respond to photoperiod and tidal phase in the* Limulus* transcriptome are novel and these findings support similar studies that found genes involved in ecological adaptation tend to be novel [24, 46].

Overall trends in transcript expression indicate a higher degree of difference in transcription occurring during high versus low tide periods than night versus day time periods. For example, there are nearly twice as many transcripts that are differentially expressed during high/low tide versus night/day (Figure 6). When comparing gene ontology term values (GO; Figures 7–9) there are five terms that vary with a minimum percent change ≥5%. When comparing these terms across conditions of night versus day we see a minimum percent change of 5% and maximum percent change of 52%. However, when looking at those same terms over high and low tidal conditions there is a minimum percent change of 36% and a maximum percent change of 198% (Figures 7–9). Taken together the results suggest a transcription profile that is dominated by tides more than by photoperiod. Deeper sequencing and statistical power will perhaps lead to the teasing apart of subtle and potentially significant changes in transcript expression.

The overall analysis of genome completeness using BUSCO was much less than that of the transcriptome (25.2% and 80.7% orthologs identified, resp.) suggesting recalcitrance by the genome and/or genome assembly to the BUSCO analysis [22]. If the genome assembly was causing the low completeness score we would expect a mapping of the transcriptome assembly to the genome assembly to be poor. However, the transcriptome maps to the genome with 88.3% coverage (Figure 3). Additionally, when the genome assembly was compared to previously reported* Limulus* genome sizes, it was found to be about half of the expected size, indicating a large, highly repetitive genome [21, 47]. Specifically, our assembly appeared to include highly frequent repetitive elements that represent a large factor of the genome. We hypothesize that it is those repeats in the genes (introns) that reduce the success of the findings universal orthologs. By contrast, the transcriptome cuts away these highly repetitive elements. Additionally, while not contributing to the low BUSCO score, other complexities with the genome structure, like the integration of mtDNA elements, may have facilitated increased collapse within the genome assembly.

Nuclear incorporation of mtDNA (NUMT) sequences can cause problems when conducting mtDNA-based studies because they can be confused with the extant version of the mitochondrial genome. To identify potential NUMTs, the* Limulus* genome assembly was searched for sequences highly similar to the extant version of the mitochondrial genome using BLASTN, which yielded two contigs. In both contigs these putative NUMT sequences were flanked by nonmitochondrial sequences including hypothetical Zinc Finger MYM-type 1 coding sequences, which are not present in any known animal mtDNA genome. The presence of these Zinc Finger sequences suggested either that the mitochondrial-like sequences were NUMTs or that there was an error which is the genome assembly causing the mtDNA sequences to be combined with the gDNA sequences. Results indicate that these sequences seem to be true NUMTs as the percent identity between the suspected NUMT sequences and the mtDNA sequences was less than 85% in either case (Figure 2). For each of these two putative NUMTs the nuclear and extant mitochondrial sequences are collinear, suggesting that these could have been derived from single translocation events and although there are two distinct mtDNA loci incorporated into two separate genomic contigs their similar levels of divergence from the extant versions may suggest a single common origin for both putative NUMTs.

The draft genome and transcriptome will begin to provide a molecular guide for a host of environmental and experimental studies. This will allow for a deeper, more thorough understanding of the behavioral and environmental impacts and challenges these animals face. These datasets are an important addition to an evergrowing aggregation of genomic and transcriptomic databases.

## 5. Conclusions

Endogenous biological rhythms are the result of complex and highly dynamic interactions between many different molecular components. When more than one rhythmic system is present in an organism, such as* Limulus*, it can be much more difficult to elucidate the most important molecules and their interactions. Here we show that* Limulus* have many genes that are considered central to other model organism's circadian clocks, but their transcripts do not seem to cycle with respect to time of day. However, there does seem to be some degree of transcript regulation with respect to tidal phase. The draft* Limulus* genome and transcriptome reported should facilitate future molecular and genetic investigations concerning biological clocks and other processes in chelicerates and other related organisms.

## Figures and Tables

**Table 1:** * Limulus* genome and transcriptome assembly statistics.

	Genome	Transcriptome
Total number of reads	620,743,244	484,378,406
Number of reads matched	573,146,494	385,105,364
Number of reads not matched	47,596,750	99,273,042
Total size of assembly	1.48 Gb	0.14 Gb
N75	2,053	1,012
N50	4,736	1,862
N25	9,523	3,497
Largest contig	68,988	23,854
Total number of contigs	744,122	93,348

**Table 2:** Comparison of *Limulus* mtDNA, JX983598.1 relative to NC_003057.1 using sites of polymorphic variance. Gene name is represented under locus. Size represents the length of the gene. Number of variable sites indicates the total number of nucleotides that differ between genomes. Number of substitutions is the combined number of transitions and transversions present at each locus. Number of frameshift mutations is the combined number of insertions and deletions present at each locus.

Locus	Size (bp)	Number of variable sites	Number of substitutions	Number of frameshift mutations
ATP6	675	19	18	1
ATP8	156	4	4	—
CYTB	1132	40	40	—
COX1	1536	36	36	—
COX2	685	18	18	—
COX3	784	17	17	—
NAD1	933	24	24	—
NAD2	1017	31	31	—
NAD3	345	13	13	—
NAD4	1338	29	29	—
NAD4L	300	12	12	—
NAD5	1714	47	47	—
NAD6	462	12	12	—
rRNA 12S	799	12	12	—
rRNA 16S	1298	26	22	4
Alanine	67	—	—	—
Arginine	63	—	—	—
Asparagine	65	1	1	—
Aspartic Acid	67	1	1	—
Cysteine	64	—	—	—
Glutamic Acid	66	1	1	—
Glutamine	66	2	2	—
Glycine	64	—	—	—
Histidine	69	—	—	—
Isoleucine	67	1	1	—
Leucine (CUN)	69	—	—	—
Leucine (UUR)	66	1	1	—
Lysine	70	—	—	—
Methionine	70	—	—	—
Phenylalanine	66	1	1	—
Proline	67	1	1	—
Serine (AGN)	73	—	—	—
Serine (UCN)	73	44	35	9
Threonine	69	—	—	—
Tryptophan	68	—	—	—
Tyrosine	67	1	1	—
Valine	69	—	—	—

**Table:** (a) Day versus Night

Contig ID	Top hit ID	Predicted gene/protein	Top hit *E*-value	*P* value	Absolute fold change	Unique gene reads (day)	Unique gene reads (night)	RPKM day	RPKM night
23185	XM_013938347	Protein trapped in endoderm-1-like	0.00*E* + 00	1.90*E* − 04	7.20*E* + 01	427	5	14.78	0.21
999	XM_013933266	DmX-like protein 2	3.39*E* − 23	3.33*E* − 07	8.37*E* + 02	1	703	0.03	25.7
84677	XM_013932736	Aldehyde dehydrogenase family 8 member A1-like	4.13*E* − 03	4.29*E* − 03	3.37*E* + 02	801	2	8.37	0.02
22396	XM_013292029	54S ribosomal protein L23	1.44*E* − 02	2.34*E* − 04	5.87*E* + 02	1	493	0.02	13.37

**Table:** (b) High versus Low Tide

Contig ID	Top hit ID	Predicted gene/protein	Top hit *E*-value	*P* value	Absolute fold change	Unique gene reads (high)	Unique gene reads (low)	RPKM high	RPKM low
132	XM_013925651	Ovostatin-like	0.00*E* + 00	1.66*E* − 11	1.68*E* + 02	50	3155	0.27	45.69
1065	XM_013933409	Thyroid peroxidase-like	0.00*E* + 00	1.10*E* − 07	3.27*E* + 02	11	1353	0.09	28.16
9027	XM_013936436	3 Beta-hydroxysteroid dehydrogenase/Delta 5->4-isomerase type 1-like	1.14*E* − 44	1.21*E* − 11	2.46*E* + 02	8	740	0.19	46.05
50915	XM_013938589	ALX homeobox protein 1-like	3.49*E* − 32	7.08*E* − 05	3.90*E* + 01	631	6	17.22	0.44

**Table 4:** Reads per kilobase per million mapped reads (RPKM) for common circadian and control genes. The absolute fold change is shown for two conditions, day/night and high/low tide. Gene name, either homologous gene title identified through BLAST or designated name given based on percent identity to similar gene variants; contig ID, contiguous sequence number given to a particular gene during assembly; RPKM values given for four individual animals (begin day high tide, day low tide, day between tides, and night high tide); absolute fold change day/night, the RPKM difference between begin day high tide and begin night high tide; absolute fold change high/low, difference between begin day high tide and begin day low tide.

Gene name	Contig ID	RPKM values				Absolute fold change day/night	Absolute fold change high/low
		Day high tide (0800)	Day low tide (1530)	Evening low tide (1800)	Night high tide (2030)		
Circadian, core							
*Period A*	12233	12.71	12.35	13.72	13.33	1.0	1.0
*Period B*	11892	6.87	4.49	5.92	6.26	1.1	1.5
*Period C*	13653	1.71	1.76	1.59	1.39	1.2	1.0
*Clock*	16515	10.65	10.97	9.84	11.28	1.1	1.0
*Cryptochrome 1*	3916	7.26	6.23	5.84	7.08	1.0	1.2
*Cryptochrome 2*	28249	4.92	6.18	6.12	6.34	1.3	1.3
*Cycle 1*	41238	1.84	0.43	1.20	1.75	1.1	4.3
*Cycle 2*	44233	0.40	0.12	0.19	0.13	3.1	3.3
*Timeless*	11686	5.28	2.85	3.43	4.09	1.3	1.9

Circadian, accessory							
*Timeout*	19500	1.31	2.44	2.50	1.15	1.1	1.9
*Double-time*	12630	6.56	6.04	5.85	7.21	1.1	1.1
*Vrille*	35732	2.24	2.17	1.73	3.68	1.6	1.0
*Casein kinase Iα*	2872	30.43	31.78	42.33	34.62	1.1	1.0
*Casein kinase IIα*	8473	20.85	25.43	19.52	24.40	1.2	1.2
*Casein kinase IIβ*	14285	16.29	16.82	15.39	17.24	1.1	1.0
*Casein kinase Iε*	2873	17.76	20.04	14.61	18.69	1.1	1.1

Circadian, regulatory							
*PDHR/CRHR*	25298	1.56	4.68	1.14	1.06	1.5	3.0

Control							
*CRP*	43896	0.36	0.38	0.36	0.36	1.0	1.1
*Tubulin*	11001	20.01	19.91	20.58	24.00	1.2	1.0
*UBQ*	243	407.00	408.00	335.00	407.00	1.0	1.0
*SDHA*	3186	124.00	139.00	142.00	129.00	1.0	1.1
*SYN*	23151	13.41	13.53	13.53	13.39	1.0	1.0
*Actin 1*	310	462.00	316.00	371.00	544.00	1.2	1.5
*Actin 2*	1424	224.00	137.00	162.00	184.00	1.2	1.6
*Actin 3*	1190	460.00	317.00	265.00	527.00	1.1	1.5
*Actin 4*	1173	366.00	346.00	379.00	381.00	1.0	1.1
*Actin 5*	334	1085.00	841.00	1169.00	1129.00	1.0	1.3
*Actin 6*	339	682.00	426.00	635.00	829.00	1.2	1.6
*Actin 7*	1232	1042.00	811.00	979.00	1046.00	1.0	1.3
